# Supplementary material for: Identification of a Sesquiterpene Lactone from Arctium lappa Leaves with Antioxidant Activity in Primary Human Muscle Cells
Source: Molecules. 2021 Mar 2;26(5):1328. doi: 10.3390/molecules26051328 (PMC7958318; doi:10.3390/molecules26051328)
Supplement: Supplementary file 1 [file molecules-26-01328-s001.zip › supplementary data diagram 1.docx]

**Identification of a sesquiterpene lactone from *Arctium lappa* leaves with antioxidant activity in primary human muscle cells**

**Nour El Khatib^1 a^, Sylvie Morel^2 a^, Gérald Hugon^1^, Sylvie Rapior^2^, Gilles Carnac^1a^ and Nathalie Saint^1 a,b^**

^1^ PhyMedExp, Univ Montpellier, CNRS, INSERM, Montpellier, France

^2^ Laboratoire de Botanique, Phytochimie et Mycologie, CEFE, Univ Montpellier, CNRS, EPHE, IRD, Univ Paul Valéry Montpellier 3, Montpellier, France

**Supplementary data**

**Diagram 1: Recapitulative scheme of purification of onopordopicrin**
