# Supplementary material for: Identification of a Sesquiterpene Lactone from Arctium lappa Leaves with Antioxidant Activity in Primary Human Muscle Cells
Source: Molecules. 2021 Mar 2;26(5):1328. doi: 10.3390/molecules26051328 (PMC7958318; doi:10.3390/molecules26051328)
Supplement: Supplementary file 1 [file molecules-26-01328-s001.zip › supplementary data table 1.docx]

**Identification of a sesquiterpene lactone from *Arctium lappa* leaves with antioxidant activity in primary human muscle cells**

**Nour El Khatib^1 a^, Sylvie Morel^2 a^, Gérald Hugon^1^, Sylvie Rapior^2^, Gilles Carnac^1a^ and Nathalie Saint^1 a,b^**

^1^ PhyMedExp, Univ Montpellier, CNRS, INSERM, Montpellier, France

^2^ Laboratoire de Botanique, Phytochimie et Mycologie, CEFE, Univ Montpellier, CNRS, EPHE, IRD, Univ Paul Valéry Montpellier 3, Montpellier, France

**Supplementary data**

**Table 1. NMR data of onopordopicrin in MeOD** (at 600 MHz for ^1^H-NMR and 150 MHz for ^13^C-NMR)

| position | δ_H_ (m, *J* in Hz) | δ_C_ | HMBC |
| --- | --- | --- | --- |
| 1 | 5.09 d (*11.2*) ^a^ | 130.5 | - |
| 2 | 2.20 m  2.30 dt (*5.6-12.6*) | 26.5 | -  C-1, C-3, C-10 |
| 3 | 2.02 m  2.64 m | 34.9 | C-2 (low), C-4 (low), C-5 (low), C-15 (low)  C-2, C-4, C-5 |
| 4 |  | 145.3 |  |
| 5 | 4.96 d (*9.7*) | 129.4 | C-3, C-7, C-15 |
| 6 | 5.20 t (*9.1*) | 78.3 | C-8 (low) |
| 7 | 3.28 m ^c^ | 53.6 | - |
| 8 | 5.12 d (*9.1*) ^a^ | 74.2 | - |
| 9 | 2.55 m  2.59 m | 49.1 | C-7 (low), C-8 (low)  C-7 (low), C-8 (low) |
| 10 |  | 132.9 |  |
| 11 |  | 137.4 |  |
| 12 |  | 172.1 |  |
| 13 | 6.19 d (*2.9*)  5.81 br s | 125.0 | C-7, C-11 (low), C-12  C-7 (low), C-12 (low) |
| 14 | 1.54 s | 16.6 | C-1, C-9, C-10 |
| 15 | 4.02 d (*13.5*)  4.25 br s ^b^ | 60.5 | C-3, C-4, C-5  C-3, C-4, C-5 |
| 16 |  | 166.2 |  |
| 17 |  | 141.6 |  |
| 18 | 4.27 br s ^b^ | 61.2 | C-16, C-17, C19 |
| 19 | 5.97 br s  6.30 br s | 125.6 | C-16, C-17 (low), C-18  C-16, C-17, C-18 |

δ value recorded in ppm from TMS (tetramethylsilane). ^1^H-(600 MHz) and ^13^C-(150 MHz) NMR measured in MeOD

^a^ and ^b^ partially overlopped.

^c^ overlopped with MeOD signal
